# Development, testing and generalizability of a standardized evaluation form for the assessment of patient-directed reports in the new final medical licensing examination in Germany

**DOI:** 10.3205/zma001467

**Published:** 2021-03-15

**Authors:** Lena Selgert, Bernd Bender, Barbara Hinding, Aline Federmann, André L. Mihaljevic, Rebekka Post, Ansgar Jonietz, John Norcini, Ara Tekian, Jana Jünger

**Affiliations:** 1 Institut für medizinische und pharmazeutische Prüfungsfragen (IMPP), Mainz, Germany; 2 Universitätsklinikum Heidelberg, Klinik für Allgemein-, Viszeral- und Transplantationschirurgie, Heidelberg, Germany; 3 "Was hab' ich?" gGmbH, Dresden, Germany; 4 SUNY Upstate Medical University, Department of Psychiatry, New York, USA; 5 University of Illinois at Chicago, College of Medicine, Illinois, USA

**Keywords:** communication, education, patient participation

## Abstract

**Background:** As doctors often fail to explain diagnoses and therapies to patients in an understandable and appropriate way, the improvement of doctor-patient communication is essential. The current medical training and examinations are focused on verbal rather than on written communication. Following the premise of “assessment drives learning”, the final medical licensing examination in Germany has been further developed by the German National Institute for state examinations in Medicine, Pharmacy and Psychotherapy (IMPP). As part of the discharge management the candidates have to prepare a report for the patient that is understandable and provides them with all important information about their stay in hospital.

**Aim:** A standardized evaluation form for formative and summative feedback has been developed and tested with regard to applicability and the assurance of test quality criteria, especially the reliability to assess the written communication skills of the students.

**Methodology:** In an expert consensus procedure, a draft for a standardized evaluation form was developed. This form was revised after an initial trial run on patient-directed reports written by students in their last year of medical studies. Afterwards twenty-one patient-directed reports were evaluated by fourteen different examiners. The reliability was tested by calculating the generalizability-coefficient and by analysing the inter-rater reliability.

**Results: **The first test on the evaluation of the patient-directed reports indicated the practicability of the application and the usefulness of the evaluation form as an instrument for assessing the written communication skills of students. The analyses of the inter-rater reliability showed that the degree of agreement in the evaluations was partly different between two groups of examiners. The calculated G-coefficient indicates a high reliability. The content validity of the evaluation form was given through the comprehensive medical expertise in the development process.

**Conclusion:** Assessing written patient-directed communication is a benefit of the newly developed last part of the medical licensing examination in Germany. Continuous formative assessment and feedback based on the evaluation form is intended to improve the written communication skills of future doctors. Furthermore, a better understanding of their diagnosis and treatment as well as a trusting relationship with their doctor may empower patients in the medical decision process and lead to fewer dismissal errors in the future. For consistent use of the evaluation form a standardized training of examiners should be implemented.

## Zusammenfassung

**Hintergrund: **Ärzt*innen erklären ihren Patient*innen Diagnosen und Therapien oft nicht ausreichend verständlich und nachvollziehbar. Eine Verbesserung der Arzt-Patienten-Kommunikation ist daher dringend geboten. Die derzeitige medizinische Ausbildung inkl. der Prüfungen konzentriert sich auf die Vermittlung von Kompetenzen in der mündlichen Kommunikation, während die schriftliche Kommunikation mit Patient*innen bislang nur wenig Beachtung findet.

Zur optimalen Vorbereitung der Medizinstudierenden auf den Berufsstart wurde das abschließende Staatsexamen des Medizinstudiums in Deutschland vom Institut für medizinische und pharmazeutische Prüfungsfragen im Sinne von „Assessment drives learning“ neugestaltet. Künftig sollen die Prüflinge als Teil des Entlassmanagements einen Bericht für die Patient*innen erstellen, der diesen in patientenverständlicher Sprache alle relevanten Informationen über ihren Krankenhausaufenthalt zugänglich macht.

**Zielsetzung: **Hierfür wurde ein standardisierter Bewertungsbogen entwickelt. Mit diesem kann ein formatives und summatives Feedback zu den Kompetenzen in schriftlicher Kommunikation gegeben werden. Die Anwendbarkeit dieses Bogens sowie die Testgütekriterien, insbesondere die Reliabilität, wurden getestet.

**Methodik: **In einem Expertenkonsensusverfahren wurde ein erster Entwurf dieses Bewertungsbogens entwickelt. Dieser Bogen wurde nach einem ersten Probelauf mit patientenverständlichen Berichten, die von Studierenden im letzten Jahr ihres Medizinstudiums verfasst wurden, überarbeitet. Anschließend wurden 21 patientenverständliche Berichte von 14 verschiedenen Prüfenden bewertet. Die Reliabilität wurde durch die Berechnung des Generalisierungskoeffizienten und durch die Analyse der Inter-Rater-Reliabilität getestet.

**Ergebnisse: **Im Rahmen der ersten Testung der Bewertung von patientenverständlichen Berichten konnte die Praktikabilität und der Nutzen des Bewertungsbogens als Instrument zur Beurteilung der spezifischen schriftlichen Kommunikationsfähigkeiten von Studierenden festgestellt werden. Die Analysen der Inter-Rater-Reliabilität zeigten, dass sich der Grad der Übereinstimmung in den Bewertungen je nach Vorkenntnissen der Prüfenden im Bereich der patientenverständlichen Kommunikation teilweise unterschied. Der berechnete G-Koeffizient weist auf eine hohe Reliabilität des Bewertungsbogens hin. Die inhaltliche Validität des Bewertungsbogens konnte durch die umfassende medizinische Expertise im Entwicklungsprozess sichergestellt werden.

**Schlussfolgerung: **Die Bewertung der Kommunikation mit Patient*innen als Teil der neu entwickelten Prüfung am Patienten oder an der Patientin bedeutet einen Zugewinn für die medizinische Ausbildung. Regelmäßige formative Prüfungen und Feedback anhand des Bewertungsbogens können im Laufe des Studiums eingesetzt werden, um die schriftlichen Kommunikationskompetenzen künftiger Ärzt*innen zu verbessern. Darüber hinaus ist zu erwarten, dass das Verstehen von Diagnosen und Therapien sowie eine vertrauensvolle Arzt-Patienten-Beziehung Patient*innen zu mehr Teilhabe am medizinischen Entscheidungsprozess befähigt. Dies könnte künftig auch Entlassungsfehlern vorbeugen. Für den einheitlichen Gebrauch des Bewertungsbogens ist die Implementierung standardisierter Prüferschulungen erforderlich.

## 1. Introduction

Following the “patient’s rights law” by the German civil law code every patient has the right to be fully informed [1]. Nevertheless, several studies show that doctors often fail to explain diagnoses and therapies to patients in an understandable and appropriate way. Therefore an improvement of doctor-patient communication is essential: 22% of patients receive incomprehensible answers to their questions and in 29% incomprehensible explanations of examination results from their doctors. As a result, 39% of patients feel left alone with their worries and fears [2]. 

Incorrect communication by medical professionals caused up to 33% of dismissal errors shown in a study between October 2012 and September 2013 [3]. Poor communication during discharge lead to medication errors, poor wound care, inadequate nutrition, rehospitalization, life-threatening situations, avoidable and unnecessary medical services and procedures as well as additional work for nursing services and increased costs for the health system. Providing sufficient and written information for patients is essential to guarantee their adherence to therapy and to implement preventive measures [2], [4], [5], [6], [7]. Especially communicating without using medical terminology is emphasized as a meaningful strategy to empower patients in their decision making process [8]. 

To improve the doctor-patient-communication of future doctors, Jünger et al. developed a longitudinal communication curriculum [9], [10]. Based on this curriculum the essential learning objectives of doctor-patient-communication can be integrated into medical training and assessment [11].

**Figure 1:**
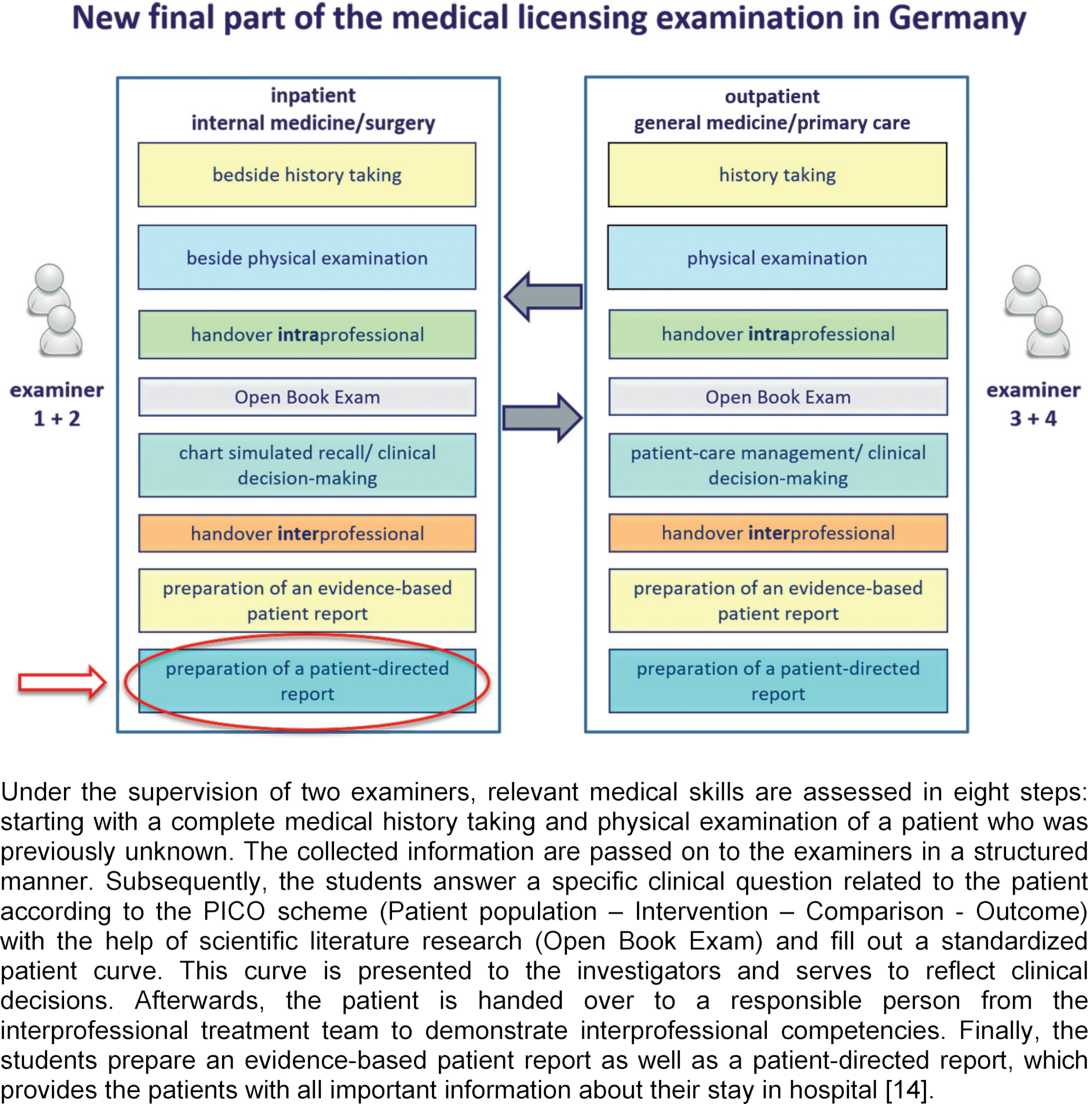
Workplace-based examination on real patients on a surgical or internal medicine ward and in the outpatient area

As assessment drives learning [12], the final part of the medical licensing examination in Germany was further developed by the German National Institute for state examinations in Medicine, Pharmacy and Psychotherapy (IMPP) to meet the needs of patients in order to optimally prepare medical students for their first day at work. Common assessments of communication skills e.g. objective structured clinical examinations, often used simulated patients and focused on verbal communication skills [13]. To increase the authenticity the new workplace-based examination demands real patients: on a surgical or internal medicine ward as well as in outpatient care (see figure 1 (Fig. 1)). As part of the improved discharge management candidates will prepare an evidence-based patient report for the post-discharge attending physician and a report for the patients themselves that is easily understandable and provides them with all important information [14]. 

Use of simple language is one of the most common strategies used by doctors, nurses and pharmacists in order to improve communication with their patients [15]. Earl et al. showed the impact of a health literacy module on the improvement of students' written patient education materials in the areas of readability, message content, computational power, statistics and concepts of patient activity. On the other hand, the simplification of medical language remained difficult [16]. Summarizing, there is already a number of international studies that have dealt with doctor-patient-communication in general and written communication in particular [17], [18], [19], [20], [21]. So far, these studies have dealt primarily with the relationship between patients' health literacy and communication, the legibility and comprehensibility of written patient information materials, as well as the benefits of written communication strategies. A larger randomized, controlled study based on an understandable patient report was already conducted with 417 patients by the initiative “Was hab’ ich?” gGmbH. The physicians at “Was hab' ich?” provided patients with an easily understandable patient report after discharge from hospital. Their study showed significant effects of the patient reports on the patients’ understanding of examination results, medication indications and prescriptions [22]. The language used in the reports was characterized by simple words, short, complete and simple sentences as well as positive language and the avoidance of medical terms. Relevant background information was provided and the text had a logical structure [23], [24]. On the basis of this collection of criteria for the preparation of a report in patient-directed language, “Was hab’ ich?” designed a template for the preparation of such reports by medical students. So far, there is no evaluation instrument for these patient-directed reports.

The aim of this study was to develop and to test a standardized evaluation form for formative and summative assessment of reports which covered the important aspects of patient-directed writing. Based on real rather than simulated patients and situations, this form had to be used individually and to cover all different kinds of settings and diseases. 

This evaluation form was based on the collection of criteria by “Was hab’ ich?” gGmbH, literature analysis and expert opinions. The applicability of the form and the test quality criteria especially the reliability were tested.

## 2. Methodology

### 2.1. Development of a standardized evaluation form for patient-directed reports

In order to assess the quality of written communication in patient-directed reports by medical students, a first draft of an evaluation form was drawn up based on a literature analysis and on a collection of important criteria for writing patient-directed by “Was hab´ ich?” gGmbH and the IMPP [23], [24]. In August 2018, a group of 27 medical experts from eight German faculties consisting of specialists in general medicine, internal medicine, anaesthesia, psychiatry, surgery, psychosomatic medicine and psychotherapy, psychologists, who were all participants in the German “Master of Medical Education” (MME) study programme, and of five students prepared a precise evaluation form in a consensus procedure: The first draft was revised first in a small group of seven experts and afterwards discussed in the whole group until a consensus was found. 

In October 2018, the evaluation form was tested on a total of ten students at the Department of Surgery of the University Hospital of Heidelberg [25]. These students were in their 4th till 6th year of medical studies. Each student wrote a patient-directed report by filling out a standardized template. This report was assessed by eleven physicians and three students who had been involved in the development of the evaluation form in August 2018. Based on these experiences the evaluation form was revised by the participants of the initiative “Was hab’ ich?”gGmbH and the IMPP.

#### 2.2. Test and revision of the evaluation form 

In January and February 2019, the revised evaluation form was tested extensively on twenty-one patient-directed reports written by students in their last (6th) year of medical studies (PJ) at the interprofessional training ward (HIPSTA) of the University Hospital of Heidelberg. At this HIPSTA, medical students and trainees from different health professions treat patients together under the supervision of medical and nursing facilitators for four weeks [26]. One part of the practical training is writing patient-directed reports in addition to the conventional discharge report to the attending physician. The PJ-students receive a structured training in patient-directed writing by the initiative “Was hab’ ich?”gGmbH. This training includes elements like the reliable recognition and avoidance of medical terms, the explanation of background information and simply structured writing. 

These twenty-one patient-directed reports were evaluated by two groups of examiners, each consisting of two physicians from the initiative “Was hab’ ich?” gGmbH. The first group used the developed evaluation form with the precise sub-items. The second group that evaluated the reports focused on the three main criteria without knowledge of the more precise sub-items [27]. 

#### 2.3. Evaluation study 

After revision of the evaluation form based on the first testing at HIPSTA, the twenty-one patient-directed reports were evaluated by a total of fourteen examiners. These examiners consisted of members from the initiative “Was hab’ ich?” gGmbH who were experienced in patient-directed writing and of physicians from the IMPP as well as general practitioners, as these are the physicians who usually receive discharge reports. The corresponding discharge report for the attending post-discharge physicians was available for comparison. The sample used for the statistical analyses included all existing valuations of all examiners on the twenty-one reports. Since not all examiners evaluated all reports, a sample of n=205 valuations resulted. The sample for the analyses on inter-rater reliability consisted of the evaluations of nine examiners, who had fully evaluated eleven of the twenty-one reports. As no personal data were evaluated, no approval of the ethics committee was needed. 

#### 2.4. Test quality criteria and statistical methods

Statistically, descriptive statistics in the form of mean values and standard deviations were first analysed separately for each of the three categories. In addition, bivariate correlations between the categories were calculated. Therefore, Pearson’s correlation coefficient was used [28].

Reliability is given when the respective instrument does not produce any measurement errors. It can be assumed if there is a high degree of intercorrelation between the individual parts of a measuring instrument. The reliability was analysed by the calculation of the generalizability coefficient [29]. Numerous measurements by the same examiners lead to an overestimation of reliability due to exercise effects when using Cronbach’s alpha [30]. In order to correct that, variance components were calculated for the factors examiner, report and background of the examiner (“Was hab’ ich?” vs. IMPP and general practitioners) regarding the reached score given in all valuations based on the generalizability theory [29], [31]. This helped to identify possible sources of measurement errors in the evaluations of the reports [32]. The relative error variance is determined based on the calculated variance components. With this relative error variance the G-coefficient can be computed. The G-coefficient estimates if the results can be transferred to the study population or if the interaction effect between the facets and the participants make the results singular to the study sample. A G-coefficient of 1 indicates that the available data and results can be perfectly generalized to all evaluations outside the study. A high value for this coefficient thus indicates high reliability [29].

The evaluation agreement in the form of inter-rater reliability [33] was calculated on the basis of a sample of nine examiners, who fully evaluated eleven of the twenty-one reports. The other examiners evaluated only some of these reports. The inter-rater reliability was handled separately for each of the three categories across the eleven reports. The analysis of inter-rater reliability was based on the coefficient Kendall W [34]. The evaluation scale of the developed form consisted of a rating scale ascending from 0 to 5 points. As at least an ordinal scale level could be assumed, Kendall W seemed to be the appropriate coefficient, unlike Fleiss Kappa for example, which requires categorical data [34]. The aim was to determine whether or not the examiners were consistent in their valuations within one evaluation category.

Furthermore, the content validity of the evaluation form was given through the comprehensive medical expertise in the development process. This means that the developed categories of the evaluation form represent exactly the content that is intended [35]. To avoid biases the examiners did not know the students who had prepared the patient-directed reports personally. 

## 3. Results

### 3.1. Development and first test of the evaluation form

This first draft contained the categories “Selection of content”, “Medical correctness”, “Report structure and syntax”, “Linguistic design” and “Grammar”. Each category could be awarded with zero to five points. Each of the five categories was weighted with ten to thirty percent. Resulting from the first test, the main evaluation criteria were summarized into three: “Selection of content and medical correctness”, “Transfer of medical language into lay language” and “Easily understandable language”. The selection of content was presented more clearly and differentiated. The three central evaluation categories were provided with specific sub-items to explain the rating categories in detail. Each category could be awarded with zero to five points. The category “Selection of content and medical correctness” was given a slightly higher weighting of forty percent. The other two categories were given a weighting of thirty percent each.

Following the first testing at HIPSTA, the previous version of the evaluation form was revised and specified. The second category was renamed “Lay language and background information”. To reduce the number of sub-items especially those of the category “selection of content and technical correctness” have been summarized from eight to five.

#### 3.2. Tested version of the evaluation form

A standardized evaluation form for patient-directed reports has been created successfully with three main evaluation categories. The percentage weighting remained forty percent for “Selection of content and medical correctness” and thirty percent each for the categories “Lay language and background information” and “Easily understandable language”. 

**Figure 2:**
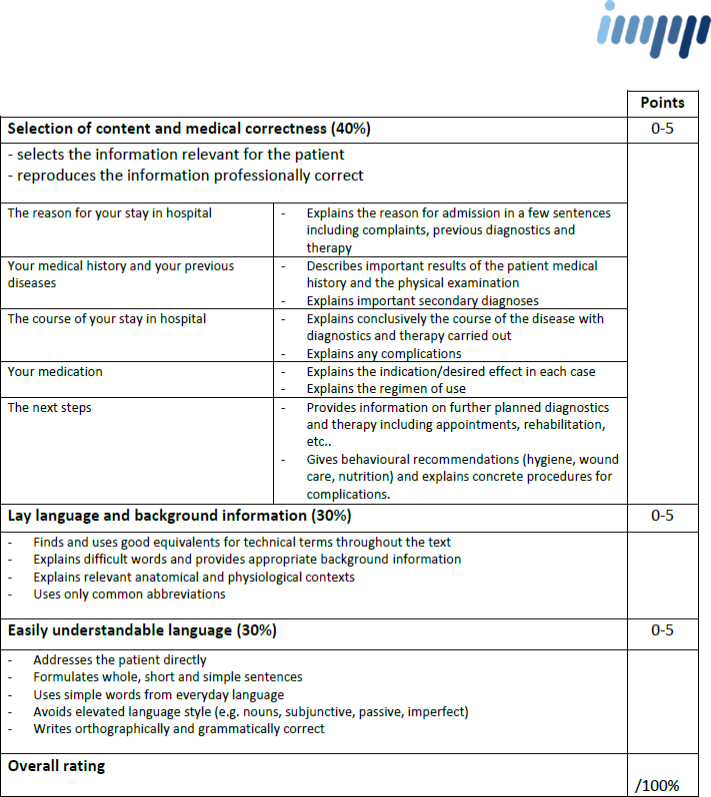
Tested evaluation form for assessment of patient-directed reports

The sub-items served to specify the content of these evaluation categories (see figure 2 (Fig. 2)).

#### 3.3. Evaluation study

##### 3.3.1. Descriptive statistics and correlations

Based on the sample of 205 individual valuations by the examiners, mean values, standard deviations and paired correlations between the three categories were calculated. 

First, an overview of mean values and standard deviations for each of the three categories of the evaluation form can be provided (see table 1 (Tab. 1)).

The mean values and standard deviations of the three categories were almost identical. This clearly showed that, on average, the same number of points was awarded across the evaluations by all fourteen examiners for the twenty-one reports. Thus, no category was rated better or worse on average.

Since the results for the descriptive statistics already suggested a possible correlation, this was checked using bivariate correlations between the three categories (see table 2 (Tab. 2)).

The results of the pairwise correlations showed medium and high positive correlations between the three categories, which were all highly significant (p-value <0.001). The strongest correlation was found between the categories “Lay language and background information” and “Easily understandable language” (r=0.61). In contrast, the correlations between “Selection of content and technical correctness” and “Lay language and background information” (r=0.45) and between “Selection of content and technical correctness” and “Easily understandable language” (r=0.31) were medium strong. 

##### 3.3.2. Reliability

**Figure 3:**
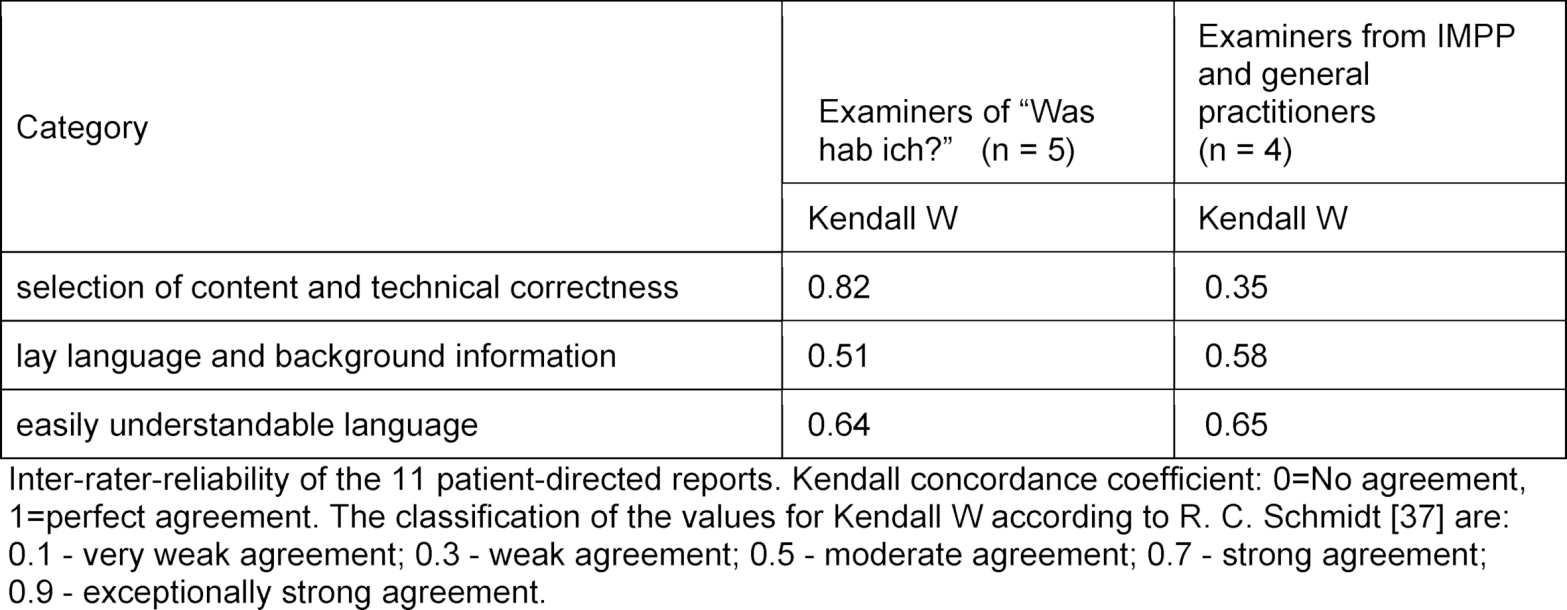
Table 3: Inter-rater reliability separately for each category across the eleven reports

The inter-rater reliability was handled separately for each of the three categories across the eleven reports. This was done for the examiners from “Was hab’ ich?” and for those from the IMPP and general practitioners, in order to compare the degree of agreement of these two groups (see table 3 (Fig. 3)). 

The degree of agreement in the first category was substantially higher for the examiners of “Was hab’ ich?” compared to the examiners from the IMPP and general practitioners. Regarding the two other categories the degree of agreement was moderate in both groups.

The **calculated G-coefficient was 0.72 based on all 205 valuations**. This rather high value is an indicator that the evaluation results of the patient-directed reports are not limited to the sample of the study, but can be transferred to evaluations outside the study. 

##### 3.3.3. Revision of the evaluation form based on the evaluation

Based on these results the evaluation form was slightly revised: The category “Lay language and background information” was specified to “Provision of background information and patient-understandable use of technical terms”. The category “Easily understandable language” was specified to “Patient-understandable language style, readability and everyday speech” to enable better differentiation of these two categories. 

The explanation of the item “your medication” was complemented with “Explains the intake schedule, gives intake instructions.” and “Indicates relevant interactions and/or adverse effects.” as well as the explanation of the behavourial recommendations of “The next steps” with “hygiene, wound care, nutrition, exercise, drinking quantity, nicotine consumption”.

## 4. Discussion

A multi-stage conception and revision process was successfully used to create a standardized evaluation form for assessment of patient-directed reports. This is a noticeable improvement of the students training in the field of doctor-patient-communication. 

It has been shown, that medical students who have undergone written communication training and regularly translate findings use better explanations than untrained students when talking to standardized patients in a simulated physician-patient contact [36]. This corresponds to the self-awareness of the students working at “Was hab’ ich”gGmbH: they are united in their opinion that written translations of doctors’ reports to patient-reports improve their ability to communicate in a way that can be understood better by patients [37]. 

In this study students had to prepare patient-directed reports in their last year of medical training. The test of a newly developed evaluation form used on twenty-one of these patient-directed reports showed the practicability as well as the usefulness of the instrument for assessing these written communication skill of the students. The individual evaluation categories represent the most important steps of writing a patient-directed report as the evaluation form has been developed by different medical experts. The implementation of specific sub-items supports the examiners in interpreting the categories.

Medium or high correlations between the three categories could be observed. Above all, the high correlation between lay language and patient-understandable language is an indicator of a good reliability of the evaluation form. In contrast, the medium-strong correlations of the content selection with lay language as well as with patient-understandable language are an indication that the evaluations of content and language can be independent of each other. Those students who, in the opinion of the examiners, used appropriate lay language in the patient reports and communicated the background information well were also able to write in language that was understandable to the patient. In contrast, an appropriate selection of content and technical correctness was not necessarily dependent on the use of lay language or easily understandable language. 

The analyses of inter-rater reliability showed that the degree of agreement in the evaluations for all three categories was partly different between the two groups of examiners. The examiners of the initiative “Was hab’ ich?” had a higher level of agreement in the category “selection of content and medical correctness” than the examiners of the IMPP and the general practitioners. This fact is an indication for different starting conditions of the examiners. This can be attributed to their different disciplinary backgrounds and especially their different previous experiences with patient-understandable language. This finding highlights the need for an uniform training of examiners on standards for writing a patient-directed report before using the evaluation form in the national licensing examination. 

The calculated G-coefficient showed that the data and results of the study can be applied to evaluations outside the study and were therefore generalizable, which showed the reliability of the evaluation form.

As the content validity of the developed evaluation form was given, the test quality criteria were mostly fulfilled. Thus, the evaluation form was a useful instrument to rate patient-directed reports with central evaluation categories. 

Some limitations include that the results were only obtained from reports written at one interprofessional training ward in Heidelberg. In the context of further research, it would be interesting to evaluate and validate the performance of assessment based on the evaluation of a larger sample of examiners and reports written on a conventional ward or in the outpatient area of different faculties. Examiner trainings on assessing patient-directed reports using the developed evaluation form should be mandatory. This training is intended to ensure a uniform assessment standard to avoid different interpretations and weightings of sub-items and to contribute to a higher assessment agreement. The inter-rater reliability of examiners who have been trained should be analysed. 

Continuous formative assessment and feedback based on the evaluation form would improve the medical training for PJ-students. As the patients are the receivers of these reports, the comparison of the assessment of the patient-directed reports by the patients themselves to the assessment by medical experts would be of great interest. Combining these two feedbacks can ensure that the patient-directed reports will deliver the important information to avoid dismissal errors in the future. 

The improvement of the verbal communication skills based on this improvement of medical training should be investigated in further studies.

## 5. Conclusion

Assessing written patient-directed communication is a benefit of the new developed last part of the medical licensing examination in Germany. Continuous formative assessment and feedback based on the evaluation form is intended to improve the written communication skills of future doctors. Furthermore, a better understanding of their diagnosis and treatment as well as a trusting relationship with their doctor may empower patients in the medical decision process and lead to fewer dismissal errors in the future. To reach this goal, clear instructions and training for writing a patient-directed report must be part of the medical curriculum. For consistent use of the evaluation form a standardized training of examiners should be implemented.

## List of abbreviations

- G-coefficient = Generalizability coefficient
- HIPSTA = Interprofessional training ward of the University Hospital of Heidelberg
- IMPP = German National Institute for state examinations in Medicine, Pharmacy and Psychotherapy
- MME = Master of Medical Education
- PJ = Elective clerkships in the final (6^th^) year
- r = Pearson’s correlation coefficient r

## Funding

This project was funded by the Bertelsmann Stiftung (duration: 1.10.2017 – 30.06.2021).

## Current professional roles of the authors

Dr. med. Lena Selgert

- Physician
- Research assistant at the German National Institute for state examinations in Medicin, Pharmacy and Psychotherapy (IMPP)

Bernd Bender

- Graduate sociologist
- Research assistant at the German National Institute for state examinations in Medicin, Pharmacy and Psychotherapy (IMPP)

Dr. phil. Barbara Hinding

- Graduate psychologist
- Research assistant at the German National Institute for state examinations in Medicin, Pharmacy and Psychotherapy (IMPP)
- Research areas: medical conversation and interprofessional communication in teaching and assessment, implementation of communication curricula in medical education and advanced training.

Aline Federmann, M.A.

- Graduate sociologist
- Research assistant at the German National Institute for state examinations in Medicin, Pharmacy and Psychotherapy (IMPP)

Prof. Dr. med. André L. Mihaljevic

- Physician and medical study program coordination (Department of General, Visceral and Transplant Surgery, Heidelberg University Hospital)
- Medical director of the interprofessional training ward in Heidelberg - (HIPSTA)
- Clinical scientist (deputy spokesman of the surgical study network CHIR-Net)

Rebekka Post

- physician working at the initiative "Was hab' ich?" gGmbH

Ansgar Jonietz

- co-founder and CEO of the initiative "Was hab' ich?" gGmbH
- computer scientist

John J. Norcini, Ph.D.

- Research Professor in the Department of Psychiatry at SUNY Upstate Medical University
- President Emeritus of FAIMER

Ara Tekian, Ph.D., MHPE

- professor, Department of Medical Education, and associate dean, International Education, University of Illinois at Chicago College of Medicine, Illinois, USA.

Prof. Dr. med. Jana Jünger, MME (Bern)

- Director of the German National Institute for state examinations in Medicine, Pharmacy and Psychotherapy (IMPP)
- Development of the post-graduation study program Master of Medical Education (MME), Germany
- Member of the MME-study program management and lecturer for the modules Assessment, Education Research and Evaluation
- Management of various programs on the implementation of communication curricula in medical training and the development of new examination formats for assessing communicative skills.

## Competing interests

The authors declare that they have no competing interests. 

## Figures and Tables

**Table 1:**
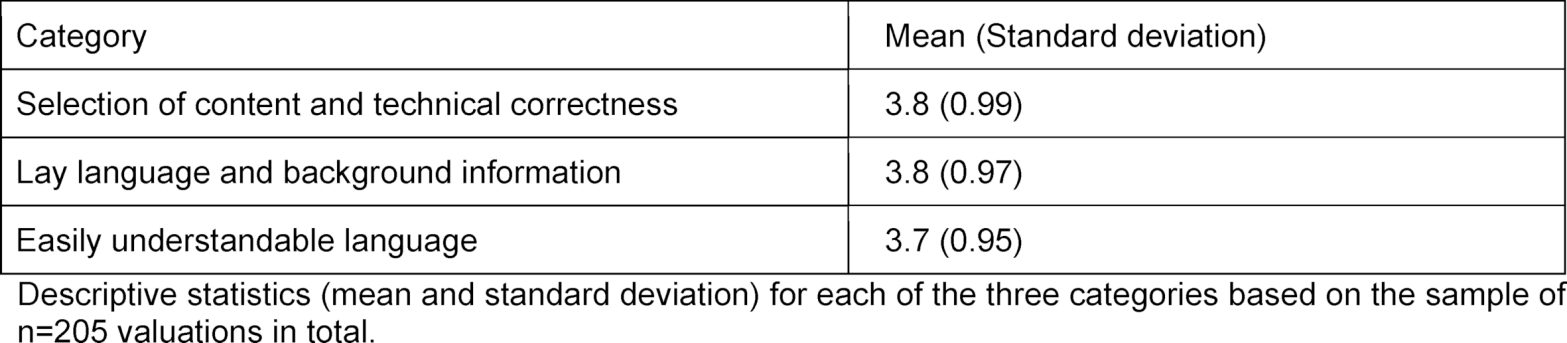
Descriptive statistics of the three evaluation categories

**Table 2:**
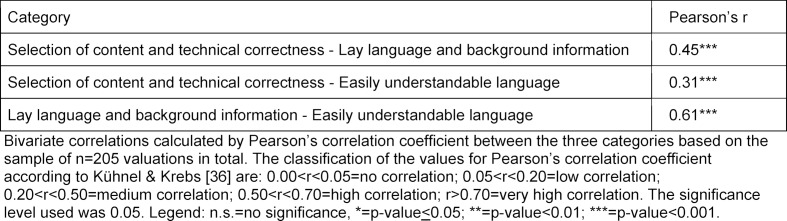
Bivariate correlations between the three categories
